# Supplementary material for: Histomolecular Validation of [18F]-FACBC in Gliomas Using Image-Localized Biopsies
Source: Cancers (Basel). 2024 Jul 18;16(14):2581. doi: 10.3390/cancers16142581 (PMC11275162; doi:10.3390/cancers16142581)
Supplement: Supplementary file 1 [file cancers-16-02581-s001.zip › Supplementary 1.pdf]

Supplementary 1: Histomolecular analysis

Fresh Frozen (FF) samples:

All samples were snap frozen in liquid nitrogen immediately after resection and stored at −80°C. For samples with enough tissue a small section of each was taken and fixated in buffered formalin with red dye. Biopsies were then stored at 37°C for a duration of 120-205 minutes, subsequently paraffin embedded and in addition to HE staining, immunohistochemistry was performed, see below. The remainder of the samples were kept frozen and 5-55 sections of 20 µm tissue or the whole sample were sectioned. For samples with enough tissue an additional cryosection (4 µm) was performed using a Thermo Cryostar NX70. The cryosections were mounted on SuperFrost Plus glass and manually HE stained according to lab-specific procedures.

Immunohistochemistry (FFPE) samples.

Paraffin sections (3 µm) were cut, mounted on Superfrost Plus glass and dried at 60°C for 60 minutes. Immunohistochemical staining was done on BenchMark Ultra fully automated tissue staining system (Ventana Medical Systems, Inc., Tucson, AZ) using validated protocols with the following antibodies:

-Mouse monoclonal IDH1-R132H antibody (clone H09, Dianova, 1:100)

-ATRX antibody (polyclonal, Sigma Aldrich, 1:500)

-Mouse monoclonal Ki-67 (clone MIB-1, Dako, 1:350)

Digital Pathology evaluation

HE-stained FFPE slides, immunohistochemistry slides and HE-stained cryosectiones were all scanned using a Hamamatsu Nanozoomer S60 and viewed using Philips Image Management System 3.3 (L4).

For IDH1 only moderate and strong cytoplasmic staining was considered positive. For ATRX only nuclear staining was considered for evaluation, and cases with more than 10% positive tumor cells were considered positive. Heterogeneous immunoreaction was observed, but only areas with highest staining were considered. AJS was blinded for the diagnosis and HE samples were evaluated as either normal, low grade glioma (LGG) or high grade glioma (HGG) based on histology alone (cellularity, mitotic activity, presence of microvascular proliferation and/or necrosis). Ki67staining was assessed by counting positive cells.

DNA extraction, bisulfite conversion and BeadChip processing:

For FF samples: DNA was extracted using Allprep DNA/RNA FFPE Kit (Qiagen, Ref. 80234). DNA yield was quantified using Qubit dsDNA HS Assay Kit (Thermo Fisher Scientific, Ref. Q32851).

For FFPE samples: DNA was extracted using QIAamp DNA FFPE Tissue Kit (Qiagen, Ref. 56404). DNA yield was quantified using Qubit dsDNA BR Assay Kit (Thermo Fisher Scientific, Ref. Q32853).

DNA quality was investigated running an in-house qPCR (fragment length 149 bp and 306 bp) to evaluate the level of DNA fragmentation.

All DNA samples were bisulfite converted using the EZ DNA Methylation Kit (Nordic BioSite, Ref. Biosite-D5001). FFPE samples were in addition processed using the Infinium HD FFPE DNA Restore Kit (Illumina, Ref. WG-321-1002). 500 ng DNA in a total sample volume of 45 µl (equals 11,1 ng/µl DNA)

was recommended as input to the bisulfite conversion reaction. Samples with DNA concentration <11,1 ng/µl were analyzed undiluted even if the total DNA input was <500 ng.

The Infinium MethylationEPIC BeadChip Kit (Illumina) was used with EPIC v1.0 BeadChip (for FF samples) and EPIC v2.0 BeadChip (for FFPE samples) to generate genome-wide DNA methylation profiles for the tumors. TECAN Freedom Evo System was used for BeadChip extension and staining. BeadChips were scanned using Illumina iScan™ System.

Unprocessed .idat-files were then uploaded to the publicly available DKFZ/Heidelberg CNS tumor classifier (https://www.molecularneuropathology.org/mnp). Methylation-based tumor classifications with prediction of class scores, indicating probability estimations of the resemblance to assigned classes in the reference cohort, were automatically generated as reports by the classifier tool using v12.5 for EPICv1 BeadChip data and 12.8 for EPICv2 BeadChip data. The reports were evaluated using the recommended threshold value ≥ 0.84.
