# Supplementary material for: Histomolecular Validation of [18F]-FACBC in Gliomas Using Image-Localized Biopsies
Source: Cancers (Basel). 2024 Jul 18;16(14):2581. doi: 10.3390/cancers16142581 (PMC11275162; doi:10.3390/cancers16142581)
Supplement: Supplementary file 1 [file cancers-16-02581-s001.zip › Supplementary 2.pdf]

Supplementary 2: Brain shift correction

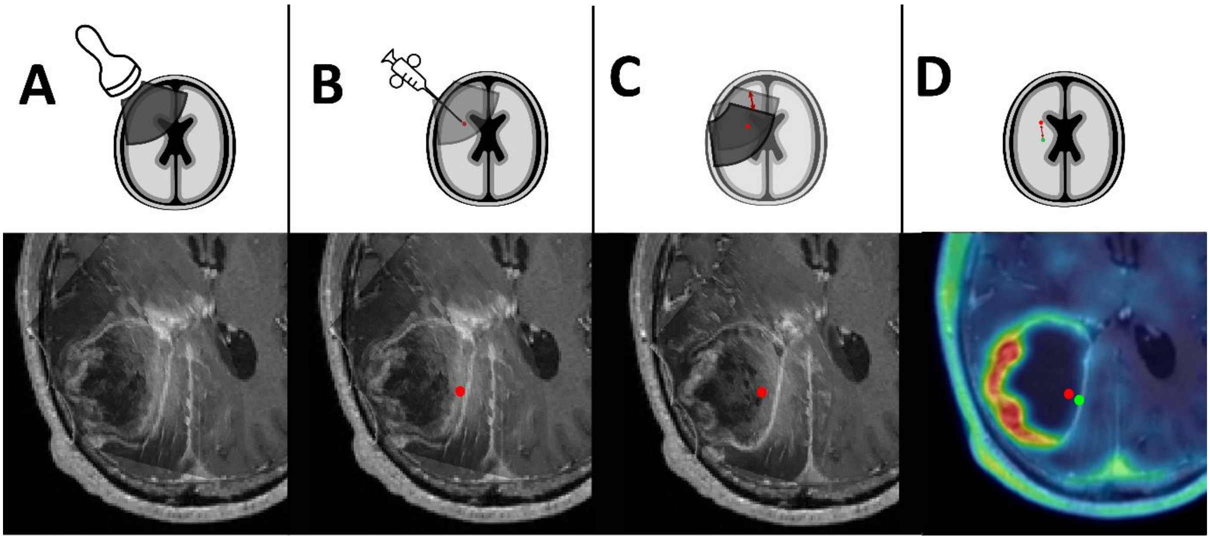

Figure S1: A: Intraoperative US images were taken during surgery. B: Biopsy coordinates (red) were registered to MR and US images. C: Post-surgery, the intraoperative US images were registered to MR images using the LC2 algorithm to account for brain shift. D: The resulting transformation matrix is applied to the biopsy coordinates to acquire brain shift corrected coordinates (green). PET TBR values were extracted from the corrected coordinates.
