# Supplementary material for: Histomolecular Validation of [18F]-FACBC in Gliomas Using Image-Localized Biopsies
Source: Cancers (Basel). 2024 Jul 18;16(14):2581. doi: 10.3390/cancers16142581 (PMC11275162; doi:10.3390/cancers16142581)
Supplement: Supplementary file 1 [file cancers-16-02581-s001.zip › Supplementary 3.pdf]

Supplementary 2

Overview of all histopathological results for image-localized biopsies

Table S1. Results for image-localized biopsies: TBR, ce-T1 contrast enhancement, classification of biopsies into LGG/HGG/non-tumor tissue from the IHC and DNA methylation analysis, classification into tumor type from DNA methylation analysis, classification of IDH1 mutation status, ATRX mutation status, and cell density from IHC analysis

ID	Final Diagnosis	Biopsy no.	Tumor Grade	Tumor Type (from DNA methylation)	TBR	ce-T1 neg (-) /pos (+)	IDH1	ATRX	Cell Density
1	Astrocytoma CNS WHO grade 2	1	LGG	n/a	0.7	-	Mutated	Lost	Low
		2	LGG	Astrocytoma	0.9	-	Mutated	Lost	Low
		3	Non-tumor tissue	n/a	0.8	-	Mutated	Lost	Low
2	Astrocytoma CNS WHO grade 2	1	n/a	n/a	1.8	-	n/a	n/a	n/a
3	Oligodendroglioma CNS WHO grade 2	1	LGG	Oligodendroglioma	0.7	-	Mutated	n/a	n/a
		2	LGG	n/a	1.0	-	Mutated	Retained	Low
		3	Non-tumor tissue	n/a	1.0	-	Wildtype	n/a	n/a
4	Astrocytoma CNS WHO grade 3	1	LGG	Astrocytoma	1.0	-	Mutated	Lost	Moderate
		2	LGG	Astrocytoma	0.8	-	Mutated	Lost	Low
		3	LGG	Astrocytoma	0.7	-	Mutated	Lost	Low
		4	LGG	Astrocytoma	0.8	-	Mutated	Lost	Low
5	Astrocytoma CNS WHO grade 3	1	LGG	Astrocytoma	2.3	-	Mutated	Lost	Moderate
		2	LGG	Astrocytoma	1.9	-	Mutated	Lost	Moderate
		3	LGG	Astrocytoma	1.8	-	Mutated	Lost	Moderate
		4	LGG	Astrocytoma	1.8	-	Mutated	n/a	n/a
6	Astrocytoma CNS WHO grade 3	1	LGG	n/a	4.2	+	Wildtype	Retained	Low
		2	LGG	n/a	3.5	+	Wildtype	Retained	Moderate
		4	Non-tumor tissue	n/a	2.7	+	Wildtype	Retained	Low
7	Oligodendroglioma CNS WHO grade 3	1	HGG	Oligodendroglioma	1.6	-	Mutated	Retained	Moderate
		2	HGG		1.5	-	Mutated	Retained	Moderate
		3	LGG	Oligodendroglioma	0.7	-	Mutated	Retained	Low
		4	HGG	Oligodendroglioma	1.7	-	Mutated	Retained	Moderate
8	Oligodendroglioma CNS WHO grade 3	1	Non-tumor tissue	n/a	3.3	+	Wildtype	Retained	Low
		2	LGG	n/a	3.1	+	Wildtype	Retained	Low
		3	LGG	Oligodendroglioma	7.4	+	Mutated	Retained	Moderate
		4	LGG	Oligodendroglioma	11.3	+	Mutated	Retained	Moderate
9	Astrocytoma CNS WHO grade 4	1	LGG	Astrocytoma	1.5	-	Mutated	Lost	Moderate
		2	LGG	Astrocytoma	2.5	-	Mutated	Lost	Low
		3	LGG	n/a	1.6	-	Mutated	Lost	Low
		4	LGG	n/a	1.9	-	Mutated	Lost	Low
10	Glioblastoma CNS WHO grade 4	1	n/a	n/a	4.1	-	n/a	n/a	n/a
		2	HGG	Glioblastoma	3.9	-	Wildtype	n/a	n/a
		3	HGG	Glioblastoma	1.7	-	Wildtype	n/a	n/a
		4	HGG	Glioblastoma	6.4	-	Wildtype	n/a	n/a
11	Glioblastoma CNS WHO grade 4	1	HGG	n/a	8.3	-	Wildtype	Retained	High
		2	HGG	Glioblastoma	6.6	+	Wildtype	n/a	n/a
		3	HGG	Glioblastoma	3.0	-	Wildtype	n/a	n/a
		4	HGG	Glioblastoma	2.2	-	Wildtype	Retained	Low
12	Glioblastoma CNS WHO grade 4	1	n/a	n/a	6.2	+	n/a	n/a	n/a
		2	HGG	Glioblastoma	0.8	-	Wildtype	n/a	n/a
		3	HGG	Glioblastoma	2.7	-	Wildtype	Retained	Moderate
		4	HGG	n/a	2.9	+	Wildtype	Retained	Moderate
13	Glioblastoma CNS WHO grade 4	1	HGG	Glioblastoma	2.7	-	Wildtype	Retained	Low
		2	HGG	Glioblastoma	13.5	+	Wildtype	n/a	n/a
14	Glioblastoma CNS WHO grade 4	1	Non-tumor tissue	n/a	1.7	+	Wildtype	Retained	Low
		2	n/a	n/a	1.8	+	Wildtype	n/a	n/a
		3	n/a	n/a	1.2	-	Wildtype	n/a	n/a
		4	n/a	n/a	1.3	-	Wildtype	n/a	n/a
15	Glioblastoma CNS WHO grade 4	1	LGG	n/a	2.0	-	Wildtype	Retained	Moderate
16	Glioblastoma CNS WHO grade 4	1	LGG	n/a	6.0	+	Wildtype	Retained	Moderate
		2	HGG	n/a	9.2	+	Wildtype	Retained	High

		3	LGG	n/a	3.1	-	Wildtype	Retained	Low
		4	Non-tumor tissue	n/a	1.1	-	Wildtype	Retained	Low
17	Glioblastoma CNS WHO grade 4	1	Non-tumor tissue	n/a	8.9	+	Wildtype	Retained	Low
		2	n/a	n/a	2.7	+	n/a	n/a	n/a
		3	n/a	n/a	4.6	+	n/a	n/a	n/a
		4	n/a	n/a	2.3	+	n/a	n/a	n/a

n/a = not available

TBR, tumor-to-background ratio; ce-T1: contrast-enhanced T1 MRI; LGG, low-grade glioma; HGG, high-grade glioma; IHC: immunohistochemical; CNS, central nervous system; WHO, world health organization
